# Neighbour Origin and Ploidy Level Drive Impact of an Alien Invasive Plant Species in a Competitive Environment

**DOI:** 10.1371/journal.pone.0155712

**Published:** 2016-05-20

**Authors:** Yan Sun, Heinz Müller-Schärer, Urs Schaffner

**Affiliations:** 1 Department of Biology/Ecology & Evolution, University of Fribourg, Chemin du Musée 10, 1700, Fribourg, Switzerland; 2 CABI, Rue des Grillons 1, 2800, Delémont, Switzerland; Austrian Federal Research Centre for Forests BFW, AUSTRIA

## Abstract

Our understanding of the potential mechanisms driving the spread and naturalization of alien plant species has increased over the past decades, but specific knowledge on the factors contributing to their increased impact in the introduced range is still urgently needed. The native European plant *Centaurea stoebe* occurs as two cytotypes with different life histories (monocarpic diploids, allo-polycarpic tetraploids). However, only tetraploids have been found in its introduced range in North America, where *C*. *stoebe* has become a most prominent plant invader. Here, we focus on the ploidy level of *C*. *stoebe* and origin of neighbouring community in explaining the high impact during the invasion of new sites in the introduced range. We conducted a mesocosm experiment under open-field conditions with the diploid (EU2x) and tetraploid (EU4x) cytotype of *Centaurea stoebe* from its native European (EU) range, and with the invasive tetraploid (NA4x) cytotype from the introduced North American (NA) range in competition with EU (old) or NA (new) neighbouring plant communities. In the presence of competition, the biomass of EU neighbouring community was reduced to a comparable level by all three geo-cytotypes of *C*. *stoebe*. In contrast, the biomass of the NA neighbouring community was reduced beyond when competing with tetraploid, but not with diploid *C*. *stoebe*. The fact that the biomass of all three geo-cytotypes of *C*. *stoebe* was correlated with the biomass of the EU neighbouring community, but not with that of the NA neighbouring community suggests that different mechanisms underlie the competitive interactions between *C*. *stoebe* and its old vs. new neighbouring communities, such as competition for the same limiting resources at home vs competition through novel allelo-chemicals or differential resource uptake strategies in the introduced range. We therefore caution to simply use the ecosystem impact assessed at home to predict impact in the introduced range.

## Introduction

Biological invasions are among the most significant components of current global environmental change, causing enormous economic and ecological losses [1, 2]. Studies on the interactions between invasive alien plant species and components of native ecosystems conducted over the past decades have greatly increased our understanding of invasion processes, but our ability to predict when, where and why invasive alien species will exert large and substantive impacts on ecosystem functioning remains limited [3, 4].

While evidence is increasing that some species traits indeed correlate with increased rates of spread of invasive alien species (e.g. Schlaepfer et al. [5]), it remains to be shown whether these or any other species traits may contribute to the dominance of an alien species when competing with a complex of resident plant species in the introduced range [6, 7]. Strong impacts have been shown to occur when invaders greatly differ in their traits from those of the natives in the invaded community [8, 9]. This, for instance, may enable invasive plant to exploit nutrients [10] or soil water [11] that residents are not able to tap in the introduced range [12]. Differences in resource acquisition between invasive alien plant species and resident species may then explain the increased impact of a plant invader in the introduced compared to its native range [13, 14]. Strauss et al. [15] reported that invasive alien grass species causing high impacts are more distantly related to native grasses than are alien but noninvasive grass species in California. Invasive alien plants may also interfere with residents due to ‘novel biochemical weapons’ released by invaders in the introduced ecosystem, ranging from changes in nutrients to changes in trophic structure, while these allelochemicals are relatively ineffective against their old neighbours with which the invader shares a co-evolutionary history [16]. Furthermore, altered biotic interactions between invasive alien plants and native mutualisms can reduce the performance of residents [17], improve nutrient availability for invaders [18] and are also reported to lead to increased reproduction of plant invaders in the introduced range [19]. The concept that recipient communities are likely to be disrupted by novel invaders may be more general. These novel interactions can result from a pre-adaptation, an inherent character of invasive alien plant species, e.g. weedy demographic traits [20, 21], which enables invaders to build up high local population densities and thereby to impose high ecosystem impact. Alternatively, increased impact has also been explained by the evolution of increased competitive ability (EICA) hypothesis, which posits that invasive species might divert available energy and resources to compete with naïve neighbours when they arrive in the introduced range without their enemies [22]. Blumenthal et al. [23] found that plants had fewer enemies in the novel environment as compared with the native environment, however, they did not find evidence of higher impact.

*Centaurea stoebe* (syn. *C*. *maculosa* Lam., Asteraceae), is a widespread, short-lived forb native to Europe that was introduced into North America as a seed contaminant some 150 years ago [24]. In Europe (EU), it exists as two cytotypes, diploids (2n = 2x = 18) and allo-tetraploids (2n = 4x = 36), which occurs in predominantly single-cytotype populations [25–28]. However, so far, only tetraploids have been recorded from its introduced North American (NA) range, despite the higher frequency of diploid populations at home, a largely sympatric and similar geographic distribution of diploid and tetraploid populations in Europe [25, 26] and evidence for multiple introductions into North America [29], which all assumes that both cytotypes have been introduced. The performance of the three *C*. *stoebe* geo-cytotypes (EU2x, EU4x and NA4x) has been well studied in the absence of competition [30]. Experimental evidence showed that the characteristics of tetraploid *C*. *stoebe*, e.g. rapid growth, perennial life cycle, higher reproduction, increased phenotypic plasticity and increased drought tolerance [30–33], as compared to diploid conspecifics may have better pre-adapted them for invading the introduced range and may also explain the absence of the diploid cytotype in North America [25, 26, 34, 35].

Evidence for post-introduction evolution in *C*. *stoebe* by genetic drift or selection is mixed. Ridenour et al. [36] found that NA *C*. *stoebe* plants grew faster than plants from EU populations, but this may not result in increased biomass of mature plants or increased reproductive output [30]. Hahn et al. [37] found earlier flowering and increased seed mass in North American tetraploids compared with European tetraploids and higher seedling emergence of tetraploid NA compared to tetraploid EU *C*. *stoebe*. Those results have been explained as a trade-off between growth and defence traits (evolution of increased competitive ability (EICA)-hypothesis) [30], suggesting post-introduction evolution in introduced tetraploid populations. Thus, present results indicate that a combination of pre-adaptation due to polyploidy and further post-introduction evolution that favoured the North American tetraploids may have contributed to their invasion success. However, whether the three geo-cytotypes differ in their impact on the resident plant community and whether the impact is affected by the origin of the competing plant community (native vs. introduced range) and thus may differentially impact the geo-cytotype’s ability as a driver of invasiveness has not been assessed simultaneously so far.

In an open-field mesocosm experiment, we set out to explore the performance of a community of old European vs. new North American plant communities when competing with one of the three geo-cytotypes of *C*. *stoebe*, and when growing in the absence of competition with *C*. *stoebe*. Specifically, we tested (1) whether the tetraploid *C*. *stoebe* are more competitive than the diploid *C*. *stoebe*, irrespective of the origin of the tetraploids and the origin of the neighbours (evidence for an inherently higher competitive ability, i.e. pre-adaptation), (2) whether tetraploid *C*. *stoebe* are particularly competitive when growing with a species mixture consisting of new, naïve plant species from the introduced range (evidence for a context-dependent pre-adaptation) or (3) whether the NA tetraploids reveal a higher impact on resident communities in general or the NA community specifically than the EU tetraploids (evidence for post-introduction evolution). Based on findings from an earlier pairwise competition study between tetraploid *C*. *stoebe* and individual EU vs. NA neighbours [38], we also assessed (4) whether the two neighbouring communities vary in their relationship between the biomass of *C*. *stoebe* and that of the neighbouring communities, and (5) whether this relationship differs among the three geo-cytotypes.

## Materials and Methods

### Ethics Statements

Swiss Federal Office for Agriculture issued a seed import permit to the CABI Centre in Delémont for our study. The study was carried out on private land. The owner of the land gave permission to conduct the study on this site. We confirm that the field study did not involve endangered or protected species.

### Study plants

We used diploid (2x) *C*. *stoebe* from native Europe (EU) and tetraploid (4x) *C*. *stoebe* from both its home (EU) and introduced range (North-Western USA, NA). Seeds of *C*. *stoebe* were collected from three EU2x, EU4x and NA4x populations each (bulk sample of 10–20 mother plants; Table 1), covering an important part of the species distribution in both ranges, i.e. E- and C-Europe, where both cytotypes are widely distributed and the most abundant area in the introduced range (Pacific NW of the USA), respectively [25, 28].

**Table 1:** Origin of *Centaurea stoebe* populations.

Geo-cytotypes of *C*. *stoebe*	Country/ state	Site code/ Region	Longitude	Latitude
**European diploid (2x)**	Romania	RO14-2x	23°41'38.50" E	46°33'96.40" N
	Austria	SAa-2x	15°43'72.95" E	48°37'09.35" N
	Hungary	H1-2x	17°76'88.91" E	46°72'20.23" N
**European tetraploid (4x)**	Hungary	H2-4x	17°44'33.97" E	47°11'65.67" N
	Hungary	HU11-4x	18°95'37.17" E	47°33'19.17" N
	Switzerland	BIERE-4x	6°33'30.16" E	46°52'41.39" N
**North American tetraploid (4x)**	Oregon/USA	Umatilla co.	118°33'16.45" W	45°29'04.98" N
	Montana/USA	Bozeman	111°01'74.26" W	45°68'54.82" N
	Oregon/USA	Dee	121°62'78.96" W	45°58'67.93" N

To assess the competitive interaction with neighbouring communities from the home vs. introduced range, seeds of seven EU and seven NA perennial plants were either collected from the field (bulk samples of 10–20 mother plants per population adjacent to a *C*. *stoebe* invaded site) or purchased from commercial suppliers in Europe and the USA for EU and NA plant species, respectively. Neighbour species were chosen among plants naturally co-occurring with *C*. *stoebe* to represent five different functional groups (Table 2). We also selected EU and NA species within functional groups with similar overall growth rates and total biomass from previous greenhouse experiment (χ^2^ = 0.28, P = 0.60 and χ^2^ = 1.38, P = 0.24 for growth rate and biomass, respectively; cf. Supplementary material: Appendix B, C and E of Sun et al. [38]). We analysed the Relative Efficiency Index (REI an indicator of mixture dynamics independent of initial plant size) among all chosen species (the data are from the previous pairwise competition experiment), and found no significant REI differences among EU species (χ^2^ = 5.38, P = 0.51) and marginally significant REI differences among NA species (χ^2^ = 12.01, P = 0.06; cf. Supplementary material: Appendix D of Sun et al. [38]). While a co-evolutionary history of the EU plants with *C*. *stoebe* is most likely at the species level, none of the seed material used in the experiment had a direct experience with *C*. *stoebe*.

**Table 2:** Species list of the native neighbour species from Europe and North America investigated in this study, their family and their functional group.

Functional group	Family	North America	Europe
**Grasses**	Poaceae	*Koeleria macrantha (Ledeb.) Schultes^1^	*Koeleria pyramidata (Lam.) Beauv^3^
	Poaceae	*Festuca idahoensis Elmer.^1^	*Festuca valesiaca Schleich.^3^
**Early season rhizomatous forbs**	Rosaceae	*Geum triflorum* Pursh^1^	*Sanguisorba minor* Scop.^1^
	Scrophulariaceae	*Penstemon procerus* Dougl.^1^	*Veronica teucrium* L.^3^
**Midseason forbs with spreading rhizomes**	Caryophyllaceae	*Monarda fistulosa L.^2^	*Dianthus carthusianorum L.^1^
**Midseason forbs with woody root crowns**	Rosaceae/ Dipsacaceae	*Potentilla arguta* Pursh^2^	*Scabiosa columbaria* L.^1^
**Late season forbs with deep-taproots**	Asteraceae	*Artemisia frigida* Willd.^*2*^	*Cichorium intybus* L.^3^

The superscript star (*) before species indicates that two individuals of this species were planted in the community, while all other species were represented by one individual only. The superscript numbers behind each species represent the source of seeds: (1) collected from field, (2) B-and-T World Seeds, Paguignan, France^#^, (3) UFA-Samen, Winterthur, Switzerland^#^.

^#^ The company guarantees for seed collection in the area of origin of each species.

### Mesocosm experimental design

The study was conducted in a fallow field next to the CABI Centre in Delémont (47°22'N, 7°19'E), Switzerland. Due to legal constraints, studies with populations from the introduced range have to be conducted under controlled conditions. We thus chose to set up the experiment in 50 L containers (49 cm surface diameter and 40 cm depth) that were sunk completely into the ground. Soil was excavated to a depth of 0.5 m, roots and rocks were removed, the soil mixed with 10 L vermiculite (Vermisol^®^, 4–8 mm grain size, VTT AG, Muttenz, Switzerland) and filled into the containers. Eleven treatments were set up by growing the EU and NA neighbouring community (10 individuals from seven EU or NA species) in the presence of the three *C*. *stoebe* geo-cytotypes (10 individuals from one of the three geo-cytotypes) and also in the absence of *C*. *stoebe*; the three geo-cytotypes of *C*. *stoebe* were also grown alone. Each treatment was replicated nine times, resulting in a total of 99 containers.

**Fig 1:**
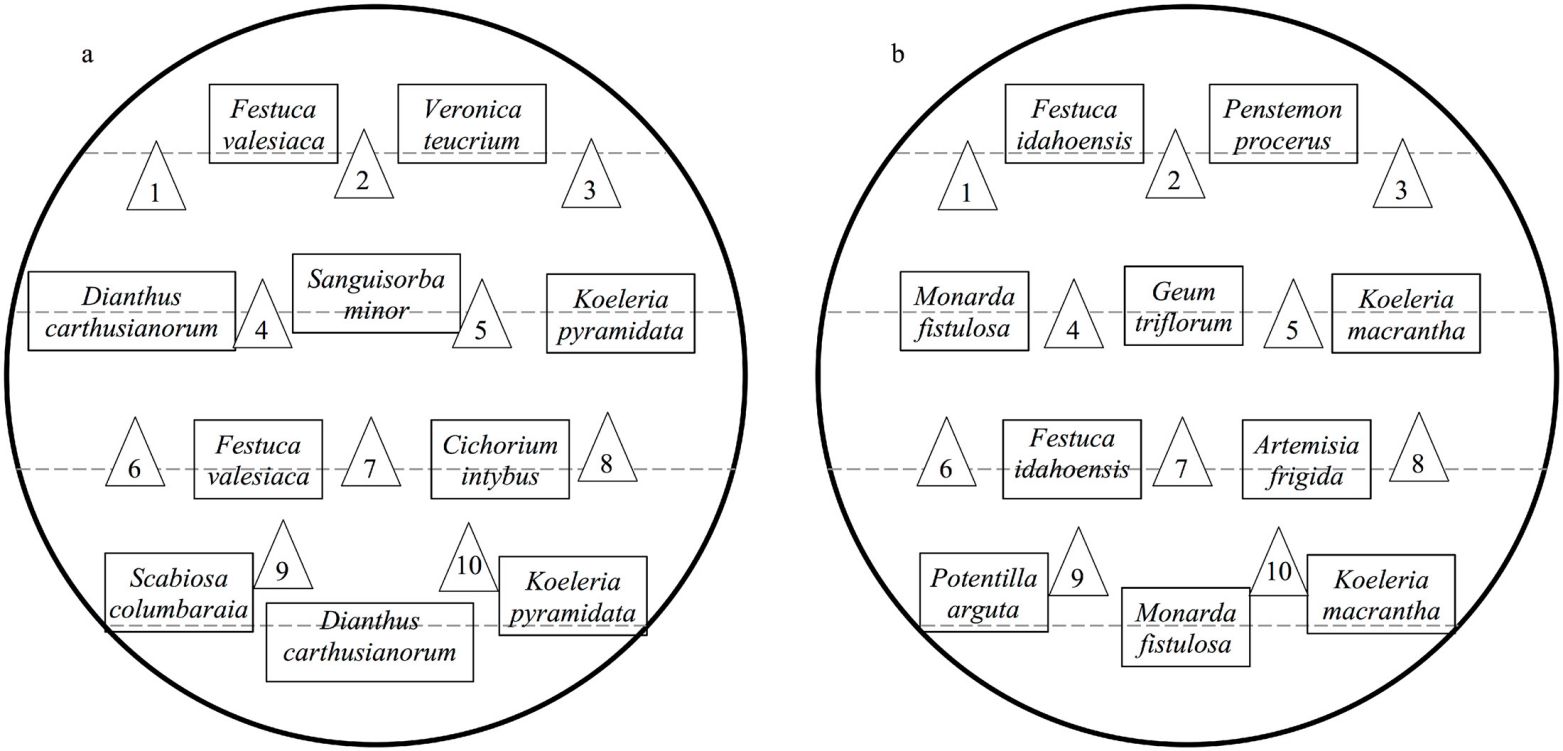
Community layout in containers. The arrangement of ten European (a) and ten North American (b) native neighbour plants and ten *Centaurea stoebe* in the interspecies competition containers; triangles are *C*. *stoebe*.

Neighbour plants from both ranges (Table 2) as well as *C*. *stoebe* from EU and NA were grown from seeds in March 2012. We sowed seeds of all species into seedling trays and grew germinated individuals in “conetainers” (2.5 cm surface diameter and 16.5 cm depth; Stuewe and Sons, Corvallis, OR) filled with commercial potting soil (Selmaterra, Eric Schweizer AG, Thun, Switzerland) mixed with sand and vermiculite (Vermisol^®^, granular form, VTT AG, Muttenz, Switzerland) in the ratio 4:2:1. The seedling trays and conetainers were kept in a CABI greenhouse and exposed to natural light condition, which was supplemented by metal halide bulbs (18h-light, 6h-dark) for seven weeks. In May 2012, we transplanted seedlings of the seven EU/NA species into the containers at an average distance of 12–14 cm. We used two individuals each of the species in the functional groups “grasses” and “midseason forb with spreading rhizomes” in a native resident community (Table 2), resulting in ten European (Fig 1a) or ten North American (Fig 1b) native neighbour plants per pot. To keep the same competition ratio (1:1) between *C*. *stoebe* and its neighbouring community as in a previous greenhouse experiment (i.e. Sun et al. [38]), ten *C*. *stoebe* seedlings randomly drawn from all populations of the same geo-cytotype were transplanted into containers with or without neighbouring community. The pattern of the spatial arrangement of the plants was the same in each container (see Fig 1). Containers were weeded weekly to avoid the emergence of seedlings of other species from the soil seed bank, and irrigation was maintained for four months (i.e. May-August).

### Data collection and statistics

Above-ground biomass of plants was harvested after six months, bagged individually (i.e. 10 and 20 bags per container for the control and competition treatments, respectively), subsequently dried to a constant weight at 60°C and weighted to an accuracy of ±0.01g.

#### Pre-adaptation vs. post-introduction evolution (hypotheses 1–3)

The biomass of neighbouring communities and *C*. *stoebe* in the absence/presence of competition was analysed using Tukey’s Honest Significant Difference (HSD) *post-hoc* comparison after one-way/two-way Analysis of Variance (ANOVA) with neighbouring community origin or/and geo-cytotypes of *C*. *stoebe* as fixed factors. We assessed the impact of three geo-cytotypes of *C*. *stoebe* in the competition containers as the comparison among aboveground biomass of neighbouring communities. For the proportion of flowering *C*. *stoebe* plants, a Generalized Linear Models (GLM) with quasi-Poisson error (due to over-dispersion, the dispersion parameter was more than 1.59 [39, 40]) was used, then ANOVA analyses were accomplished by comparing the model fits with model deleted independent variables in which we specified test = “F”. Multiple comparisons among factors were done using a *t*-test based on the best model and pairwise comparisons within the factor were done using Tukey Contrasts. We thus assessed the early relative reproductive capacity of three geo-cytotypes of *C*. *stoebe* in the competition containers as the comparison among the proportion flowering of *C*. *stoebe*.

#### Mechanisms underlying impact by *C*. *stoebe* (hypotheses 4 & 5)

Mixed-effects regression models were used to analyse the correlation between log10- transformed biomass of neighbouring communities and *C*. *stoebe* in competition containers. Origin of neighbouring communities was also included as fixed effects in a combined analysis of data sets. As to the random structure, we compared a random intercept and slope model and a random intercept model using geo-cytotype as a factor, and used the likelihood ratio test from maximum likelihood (ML) fits for significance. They indicated no difference between the common slope and the slopes of each of the geo-cytotype (P > 0.1). Eventually, model-II simple linear regression (geometric mean regression) using standard major axis (SMA) method [41] was used because both x and y variables were measurements, to compute the relationship between log10- transformed biomass of neighbouring communities and *C*. *stoebe*. All analyses were performed using R statistical software, version 3.0.1 (R Development Core Team, 2013).

The experimental design did not allow testing *C*. *stoebe* origin against the number of *C*. *stoebe* populations. Thus while our approach allowed a considerable statistical power despite the low number of populations within *C*. *stoebe*, a significant *C*. *stoebe* origin effect would have to be interpreted with caution since the statistical analysis does not distinguish between among-population and within-population effects.

## Results

### In the absence of competition

**Fig 2:**
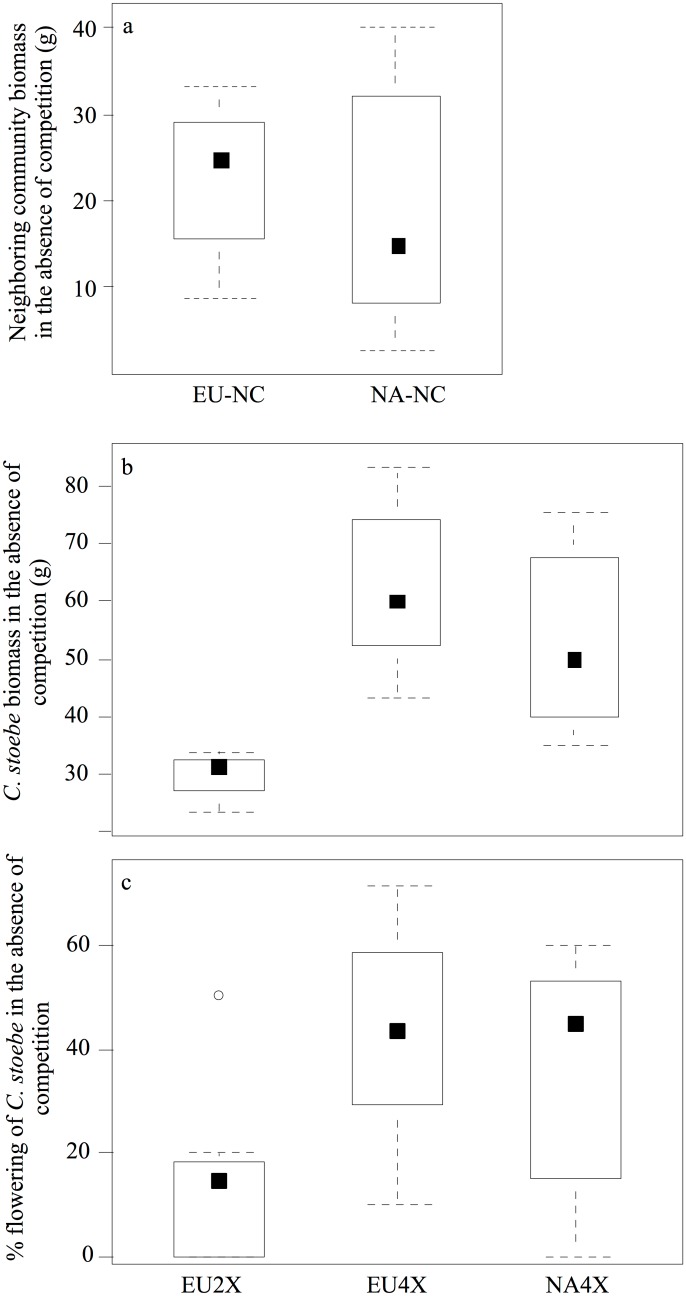
Performance of neighbouring community and *C*. *stoebe* in the absence of competition. In the absence of competition, biomass (g dry weight) of European and North American neighbouring community (EU-NC and NA-NC, respectively) (a), and biomass (b) of and proportion flowering (c) of European diploid (EU2x), European tetraploid (EU4x) and North American tetraploid (NA4x) *Centaurea stoebe*.

At the end of the experiment, the biomass of the EU and NA neighbouring community did not differ when grown without competition (F_1, 16_ = 0.37, P = 0.55; Fig 2a).

In the absence of competition, the biomass of *C*. *stoebe* differed significantly among the three geo-cytotypes (F_2, 24_ = 6.21, P = 0.01; Fig 2b). Specifically, the biomass of EU2x *C*. *stoebe* was lower than that of EU4x and NA4x *C*. *stoebe* (Tukey’s HSD, P = 0.002 & P = 0.01, respectively), while no difference was found between the two 4x *C*. *stoebe* (Tukey’s HSD, P = 0.39; Fig 2b). Percent flowering of *C*. *stoebe* also significantly differed among the three geo-cytotypes (Resid. Deviance = 4.72, F = 3.84, P = 0.04, Fig 2c), with that of EU 2x *C*. *stoebe* being lower than that of the 4x *C*. *stoebe* (Tukey Contrasts, P = 0.01 & P = 0.04, respectively; Fig 2c). No difference was found between EU4x and NA4x *C*. *stoebe* (Tukey Contrasts, P = 0.82, Fig 2c).

### In the presence of competition

**Fig 3:**
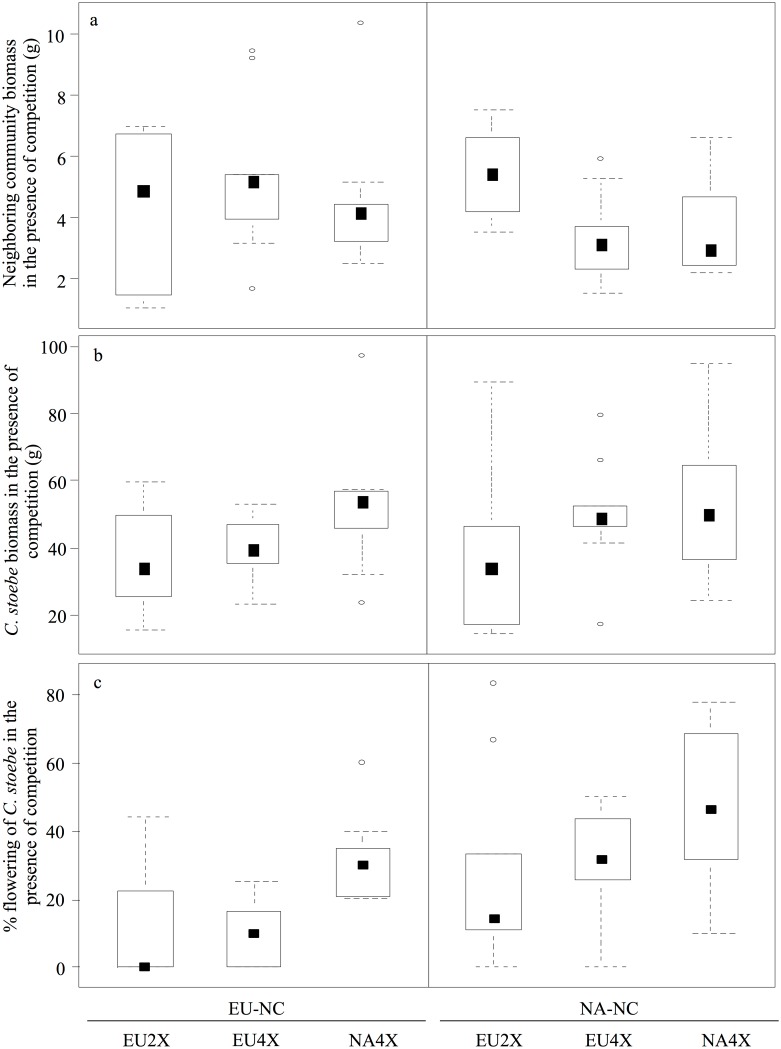
Performance of neighbouring community and *C*. *stoebe* in the presence of competition. In the presence of competition, biomass (g dry weight) of European and North American neighbouring community (EU-NC and NA-NC, respectively) (a), and biomass (b) of and proportion flowering (c) of European diploid (EU2x), European tetraploid (EU4x) and North American tetraploid (NA4x) *Centaurea stoebe*.

In the presence of competition, the biomass of EU and NA neighbouring community did not consistently differ at the end of the experiment (F_1, 48_ = 0.67, P = 0.42; Fig 3a), but was approximately four to five times smaller than when grown without *C*. *stoebe*. Also, the biomass of EU neighbouring community did not differ when grown with the three different *C*. *stoebe* geo-cytotypes (Tukey's HSD, P = 0.57 & P = 0.95, respectively). In contrast, the biomass of NA neighbouring community was significantly lower when grown with the tetraploid cytotype than with the EU2x *C*. *stoebe* (Tukey's HSD, P = 0.01 & P = 0.04, respectively; Fig 3a), while it was similar when grown with EU4x and NA4x *C*. *stoebe* (Tukey's HSD, P = 0.88). Moreover, the biomass of EU neighbouring community was significantly higher than that of NA neighbouring community when grown with tetraploid *C*. *stoebe* (i.e. EU4x and NA4x; Tukey’s HSD, P = 0.04; Fig 3a). In contrast, biomass of EU neighbouring community was similar to that of NA neighbouring community when grown with diploid *C*. *stoebe* (Tukey’s HSD, P = 0.17; Fig 3a).

Overall, the biomass of the three geo-cytotypes of *C*. *stoebe* did not differ, nor did they differ when competing with EU or NA neighbouring community biomass (F_2, 48_ = 1.91, P = 0.16 and F_1, 48_ = 1.07, P = 0.31 for geo-cytotypes and NC origins, respectively; Fig 3b). However, the proportion of flowering *C*. *stoebe* differed both among geo-cytotypes as well as between neighbouring community origins (Resid. Deviance = 10.2, F = 3.53, P = 0.04 and Resid. Deviance = 7.51, F = 8.88, P = 0.005 for geo-cytotypes and NC origins, respectively; Fig 3c). Specifically, a significantly higher proportion of NA4x *C*. *stoebe* flowered when competing with neighbouring community than the two EU cytotypes (Tukey Contrasts, P = 0.03 & P = 0.04, for EU2x and EU4x respectively), while EU2x and EU4x *C*. *stoebe* did not differ in their flowering proportion (Tukey Contrasts, P = 0.99; Fig 3c). Moreover, *C*. *stoebe* had a higher flowering proportion when competing with NA neighbouring community than with EU neighbouring community (Tukey Contrasts, P = 0.004; Fig 3c).

### Interactions between *C*. *stoebe* and its neighbouring communities

**Fig 4:**
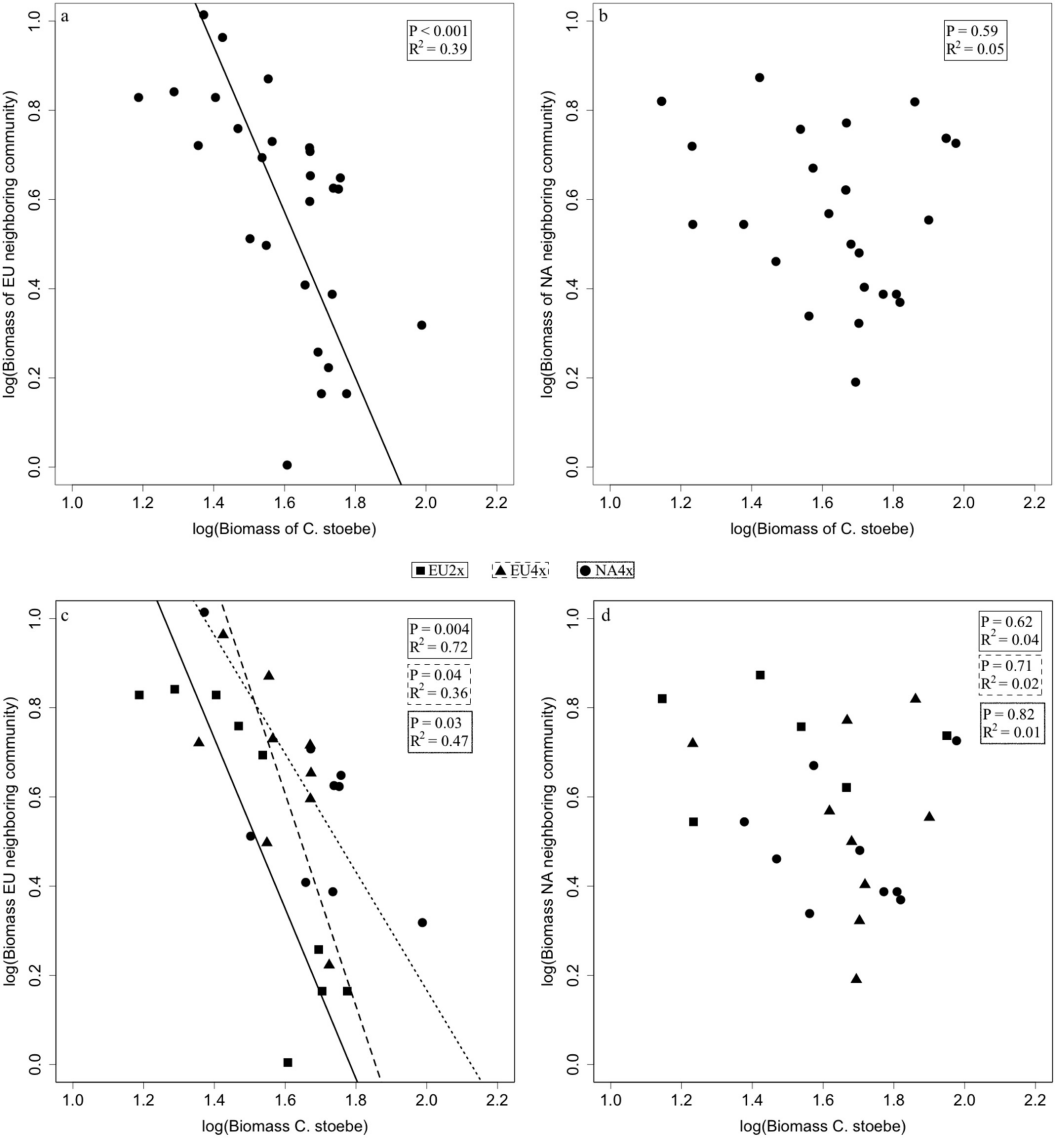
Relationships between *C*. *stoebe* and its neighbouring community. Relationship between the log10-transformed biomass of the pooled geo-cytotypes of *Centaurea stoebe* and that of European (a) and North American (b) neighbouring community in competition pots; and relationship between biomass of single geo-cytotypes of *Centaurea stoebe* and that of European (c) and North American (d) neighbouring community in competition pots. Squares and solid lines represent EU2x, triangles and dashed lines represent EU4x, and circles and dotted lines represent NA4x.

In the competition containers, biomass of EU neighbouring community explained a highly significant and substantial amount of the variation in biomass of *C*. *stoebe* (R^2^ = 0.39, Δlog-likelihood = 4.91, P < 0.001; Fig 4a), but NA neighbouring community did not explain variation in biomass of *C*. *stoebe* (R^2^ = 0.05, Δlog-likelihood = -13.96, P = 0.48; Fig 4b). The relationship between EU neighbouring community and the three geo-cytotypes of *C*. *stoebe* remained significant when analysing the geo-cytotypes separately (EU2x: R^2^ = 0.72, Δlog-likelihood = 2.96, P = 0.004; EU4x: R^2^ = 0.36, Δlog-likelihood = 3.56, P = 0.04; NA4x: R^2^ = 0.47, Δlog-likelihood = 4.66, P = 0.04; Fig 4c). Similarly, biomass of NA neighbouring community did not explain a significant amount of variation in biomass of all three ploidy *C*. *stoebe* (EU2x: R^2^ = 0.04, Δlog-likelihood = -0.34, P = 0.62; EU4x: R^2^ = 0.02, Δlog-likelihood = -5.63, P = 0.71; NA4x: R^2^ = 0.01, Δlog-likelihood = -1.83, P = 0.83; Fig 4d).

## Discussion

Our results suggest that a context-dependent pre-adaptation of the tetraploid *C*. *stoebe* rather than post-introduction evolution in North American tetraploids contributes to the high impact of North American tetraploid *C*. *stoebe* in North America (cf. our hypotheses 2 & 3 of the Introduction). Tetraploids reduced the biomass of the resident community more significantly than diploids, but this difference was only observed when competing with NA natives. Since the growth rate of the EU vs. NA neighbouring community in the non-competition containers did not differ consistently, our results indicate that tetraploid *C*. *stoebe* inflict an over-proportional impact on NA neighbouring community compared to EU neighbouring community (hypotheses 1 & 2). The fact that the biomass of all three *C*. *stoebe* geo-cytotypes was correlated with the biomass of the EU neighbouring community, but not with that of the NA neighbouring community (hypotheses 4 & 5), suggests an inherently different mechanism underlying the competitive interactions between *C*. *stoebe* and its old neighbouring community in Europe vs. *C*. *stoebe* and its new neighbouring community in North America. This pattern is unlikely to be influenced by individual neighbouring species. While a marginally significant variation in impact was found among the NA species used in this study (see the REI results in the M&M), no significant variation in impact was found among sixteen NA species (including the species used in this study) in a previously conducted pairwise competition experiment ([38]); also, EU species did not differ in impact neither in this study nor in the previously conducted experiment ([38]).

Based on the results of a short-term greenhouse study, Sun et al. [38] proposed that the relationship between the biomass of the invader and the biomass of resident plant species might shed light on the type of interspecific interaction in operation. They found a significant negative linear relationship between the biomass produced by *C*. *stoebe* and that of its old neighbours at home that share a co-evolutionary history with the invader, suggesting that they compete for the same limiting resources. In contrast, the biomass of *C*. *stoebe* explained very little of the variation in biomass of the naïve neighbours in the introduced range, indicating that the impact is driven by other forms of negative interactions (cf. below). Similarly, we also found significant negative relationships between biomass of *C*. *stoebe* and that of EU neighbouring community for all three geo-cytotypes. In contrast, the lack of a significant relationship between biomass of *C*. *stoebe* and that of North American neighbouring community suggests that the negative interactions of all three geo-cytotypes of *C*. *stoebe* with its naïve neighbours was driven by other mechanisms than resource competition, such as by exploitation of resources that are not utilized by neighbours [38, 42] or by interference competition [43, 44]. This indicates that the different impact types of *C*. *stoebe* in its home vs. introduced range are more likely driven by the native community origin (hypothesis 4) rather than the geo-cytotypes of *C*. *stoebe* (hypothesis 5).

Despite the fact that both diploid and tetraploid *C*. *stoebe* appeared to interact with NA native communities differently than with EU native communities, the diploids which did not become invasive in North America [25], did not inflict an increased impact on NA native communities. In the absence of competition, the tetraploid *C*. *stoebe* grew significantly larger than diploid *C*. *stoebe*. However, it is not clear whether this may explain the increased impact of tetraploid *C*. *stoebe* on NA neighbouring community, since in the competition containers all *C*. *stoebe* geo-cytotypes reached a comparable biomass at the end of the experiment.

In our experiment we observed no evidence for post-introduction evolutionary change in impact of *C*. *stoebe*. On the other hand, in the presence of competition a significant increase in early reproductive capacity in NA4x *C*. *stoebe* populations was found, compared to the two cytotypes from the native range, and no differences were observed between the two cytotypes within Europe. In line with the study by Callaway et al. [19], but in contrast to that by Hahn et al. [37], we observed a higher early reproductive capacity of NA4x *C*. *stoebe* only in the competition treatment.

It should be noted though that we only tested a limited amount of populations of *C*. *stoebe* of the three geo-cytotypes in the experiment. Nevertheless, the increased impact of 4x *C*. *stoebe* (no differences between EU4x and NA4x) on NA plant species as detected in this experiment is unlikely to be driven by post-introduction evolution of increased impact of *C*. *stoebe*, as we found no significant difference in impact between EUU4x and NA4x *C*. *stoebe*. This pattern is well in line with earlier results of a greenhouse experiment [38] as well as with a field study [19].

The results of this study indicate that the invasive tetraploid cytotype of *C*. *stoebe* not only harbours traits that increase its population build-up [30, 32, 45], but also traits that inflict an inherently higher impact on naïve neighbours than diploid *C*. *stoebe*. Our study also proposes that assessments of impact of alien plant species preferentially should be conducted in a competitive environment using the naïve neighbours from the introduced range. Up to date, only a small proportion of studies on invasive plant species specifically addressed impact by taking a biogeographic approach [3, 46]. Similarly to our findings, Callaway et al. [47] showed that *Rhaponticum repens* (L.) Hidalgo (formerly *Acroptilon repens* (L.) DC.), a highly invasive forb in North America, had stronger competitive effects against native North American species than against species native to Uzbekistan, the home range of *R*. *repens*. The effects of leachates collected from *R*. *repens* roots were weak but more negative on species from North America than on species from Uzbekistan. Canopies of *Prosopis juliflora*, a native leguminous tree of the New World but an invader in many other regions, had facilitative effects on the diversity of other species in its native range. However, in India and on Hawaii, USA, where *P*. *juliflora* is an aggressive invader, canopy effects were consistently and strongly negative on species richness [48]. *Chromolaena odorata* (L.) R. M. King and H. Robinson had stronger competitive effects on native species from China (the invaded range) than on *native* species from Mexico (the native range of *C*. *odorata*) under high nutrient conditions, and the germination and growth of species native to China was far more inhibited by extracts from *C*. *odorata* leaves than those of species native to Mexico [49]. Hence, while still more studies comparing the impact of an invasive plant at home and away are needed, there is a growing body of evidence that the biogeographic origin of species does indeed matter for understanding the impacts of invasive plant species (cf. Richardson and Ricciardi [50] for the recent controversy in invasion science on “does origin matter”).

## References

[1] Gurevitch J, Fox GA, Wardle GM, Inderjit, Taub D. Emergent insights from the synthesis of conceptual frameworks for biological invasions. Ecology Letters. 2011;14(4):407–18. 21513009 10.1111/j.1461-0248.2011.01594.x

[2] Pimentel D, Zuniga R, Morrison D. Update on the environmental and economic costs associated with alien-invasive species in the United States. Ecological Economics. 2005;52(3):273–88.

[3] Levine JM, Vilà M, Antonio CMD, Dukes JS, Grigulis K, Lavorel S. Mechanisms underlying the impacts of exotic plant invasions. Proceedings of the Royal Society of London Series B: Biological Sciences. 2003;270(1517):775–81. 12737654 10.1098/rspb.2003.2327 PMC1691311

[4] Hulme PE, Pyšek P, Jarošík V, Pergl J, Schaffner U, Vilà M. Bias and error in understanding plant invasion impacts. Trends in ecology & evolution. 2013;28(4):212–8. 23153723 10.1016/j.tree.2012.10.010

[5] Schlaepfer DR, Glättli M, Fischer M, van Kleunen M. A multi-species experiment in their native range indicates pre-adaptation of invasive alien plant species. New Phytologist. 2010;185(4):1087–99. 10.1111/j.1469-8137.2009.03114.x 19968796

[6] Lockwood JL, Hoopes MF, Marchetti MP. Invasion ecology: John Wiley & Sons; 2013.

[7] Ordonez A. Global meta-analysis of trait consistency of non-native plants between their native and introduced areas. Global ecology and biogeography. 2014;23(3):264–73.

[8] Vitousek PM. Biological invasions and ecosystem processes: towards an integration of population biology and ecosystem studies Ecosystem Management: Springer; 1996 p. 183–91.

[9] Cleland EE. Trait divergence and the ecosystem impacts of invading species. New Phytologist. 2011;189(3):649–52. 10.1111/j.1469-8137.2010.03607.x 21223282

[10] Davis MA, Grime JP, Thompson K. Fluctuating resources in plant communities: a general theory of invasibility. Journal of Ecology. 2000;88(3):528–34.

[11] Enloe SF, DiTomaso JM, Orloff SB, Drake DJ. Soil water dynamics differ among rangeland plant communities dominated by yellow starthistle (*Centaurea solstitialis*), annual grasses, or perennial grasses. Weed Science. 2004;52:929–35.

[12] Vilà M, Weiner J. Are invasive plant species better competitors than native plant species?–evidence from pair-wise experiments. Oikos. 2004;105(2):229–38.

[13] Pattison RR, Goldstein G, Ares A. Growth, biomass allocation and photosynthesis of invasive and native Hawaiian rainforest species. Oecologia. 1998;117(4):449–59. 28307669 10.1007/s004420050680

[14] Funk JL, Vitousek PM. Resource-use efficiency and plant invasion in low-resource systems. Nature. 2007;446(7139):1079–81. 17460672 10.1038/nature05719

[15] Strauss SY, Webb CO, Salamin N. Exotic taxa less related to native species are more invasive. Proceedings of the National Academy of Sciences. 2006;103(15):5841–5. 10.1073/pnas.0508073103 PMC1421337 16581902

[16] Callaway RM, Aschehoug ET. Invasive plants versus their new and old neighbors: A mechanism for exotic invasion. Science. 2000;290(5491):521–3. 11039934 10.1126/science.290.5491.521

[17] Callaway RM, Cipollini D, Barto K, Thelen GC, Hallett SG, Prati D, et al Novel weapons: invasive plant suppresses fungal mutualists in America but not in its native Europe. Ecology. 2008;89(4):1043–55. 18481529 10.1890/07-0370.1

[18] Tharayil N, Bhowmik P, Alpert P, Walker E, Amarasiriwardena D, Xing B. Dual purpose secondary compounds: phytotoxin of *Centaurea diffusa* also facilitates nutrient uptake. New Phytologist. 2009;181(2):424–34. 10.1111/j.1469-8137.2008.02647.x 19121037

[19] Callaway R, Waller L, Diaconu A, Pal R, Collins A, Müller-Schärer H, et al Escape from competition: neighbors reduce C. stoebe performance at home but not away. Ecology. 2011. 10.1890/11-0518.1 22352160

[20] Baker HG. The evolution of weeds. Annual review of ecology and systematics. 1974:1–24.

[21] Leibold MA, Holyoak M, Mouquet N, Amarasekare P, Chase JM, Hoopes MF, et al The metacommunity concept: a framework for multi-scale community ecology. Ecology letters. 2004;7(7):601–13.

[22] Coley PD, Bryant JP, Chapin FS Iii. Resource availability and plant antiherbivore defense. Science(Washington). 1985;230(4728):895–9. 17739203 10.1126/science.230.4728.895

[23] Blumenthal D, Mitchell CE, Pyšek P, Jarošık V. Synergy between resource availability and pathogen release in plant invasion. Proceedings of the National Academy of Sciences USA. 2009;106:7899–904. 10.1073/pnas.0812607106 PMC2674393 19416888

[24] Roché CT, Roché J, Ben F. Meadow knapweed invasion in the Pacific Northwest, USA and British Columbia, Canada. Northwest Science. 1991;65(1):53–61.

[25] Treier UA, Broennimann O, Normand S, Guisan A, Schaffner U, Steinger T, et al Shift in cytotype frequency and niche space in the invasive plant *Centaurea maculosa*. Ecology. 2009;90(5):1366–77. 19537556 10.1890/08-0420.1

[26] Mráz P, Bourchier RS, Treier UA, Schaffner U, Müller-Schärer H. Polyploidy in phenotypic space and invasion context: a morphometric study of *Centaurea stoebe* sl. International Journal of Plant Sciences. 2011;172(3):386–402.

[27] Mráz P, Španiel S, Keller A, Bowmann G, Farkas A, Šingliarová B, et al Anthropogenic disturbance as a driver of microspatial and microhabitat segregation of cytotypes of *Centaurea stoebe* and cytotype interactions in secondary contact zones. Annals of botany. 2012;110(3):615–27. 10.1093/aob/mcs120 22730023 PMC3400448

[28] Mráz P, Garcia-Jacas N, Gex-Fabry E, Susanna A, Barres L, Müller-Schärer H. Allopolyploid origin of highly invasive *Centaurea stoebe* sl (Asteraceae). Mol Phylogen Evol. 2012;62(2):612–23. 10.1016/j.ympev.2011.11.006 22126902

[29] Marrs RA, Sforza R, Hufbauer RA. Evidence for multiple introductions of *Centaurea stoebe* micranthos (spotted knapweed, Asteraceae) to North America. Molecular Ecology. 2008;17(19):4197–208. 19378400 10.1111/j.1365-294x.2008.03903.x

[30] Henery ML, Bowman G, Mráz P, Treier UA, Gex-Fabry E, Schaffner U, et al Evidence for a combination of pre-adapted traits and rapid adaptive change in the invasive plant Centaurea stoebe. Journal of Ecology. 2010;98(4):800–13.

[31] Mráz P, Tarbush E, Müller-Schärer H. Drought tolerance and plasticity in the invasive knapweed Centaurea stoebe sl (Asteraceae): effect of populations stronger than those of cytotype and range. Annals of botany. 2014:mcu105. 10.1093/aob/mcu105 PMC4111397 24918204

[32] Hahn MA, van Kleunen M, Müller-Schärer H. Increased phenotypic plasticity to climate may have boosted the invasion success of polyploid Centaurea stoebe. PloS one. 2012;7(11):e50284 10.1371/journal.pone.0050284 23185598 PMC3502303

[33] Broz AK, Manter DK, Bowman G, Müller-Schärer H, Vivanco JM. Plant origin and ploidy influence gene expression and life cycle characteristics in an invasive weed. Bmc Plant Biology. 2009;9. 10.1186/1471-2229-9-33 PMC2670832 19309502

[34] Broennimann O, Treier UA, Müller-Schärer H, Thuiller W, Peterson AT, Guisan A. Evidence of climatic niche shift during biological invasion. Ecology Letters. 2007;10(8):701–9. 17594425 10.1111/j.1461-0248.2007.01060.x

[35] Petitpierre B, Kueffer C, Broennimann O, Randin C, Daehler C, Guisan A. Climatic niche shifts are rare among terrestrial plant invaders. Science. 2012;335(6074):1344–8. 10.1126/science.1215933 22422981

[36] Ridenour WM, Vivanco JM, Feng Y, Horiuchi J-i, Callaway RM. No evidence for trade-offs: *Centaurea* plants from America are better competitors and defenders. Ecological Monographs. 2008;78(3):369–86.

[37] Hahn MA, Lanz T, Fasel D, Müller-Schärer H. Increased seed survival and seedling emergence in a polyploid plant invader. American journal of botany. 2013;100(8):1555–61. 10.3732/ajb.1200540 23935112

[38] Sun Y, Collins AR, Schaffner U, Müller-Schärer H. Dissecting impact of plant invaders: do invaders behave differently in the new range? Ecology. 2013;94(10):2124–30. 24358696 10.1890/12-1910.1

[39] Zuur A, Ieno EN, Walker N, Saveliev AA, Smith GM. Mixed effects models and extensions in ecology with R: Springer Science & Business Media; 2009.

[40] Zuur A, Ieno E, Smith G. Analysing ecological data. Statistics for biology and health series. Springer, New York; 2007.

[41] Sokal R, Rohlf F. Biometry: the principles and practices of statistics in biological rResearch: WH Freeman and Co; 2011.

[42] Ortega YK, Pearson DE, Waller LP, Sturdevant NJ, Maron JL. Population-level compensation impedes biological control of an invasive forb and indirect release of a native grass. Ecology. 2012;93(4):783–92. 22690629 10.1890/11-0750.1

[43] Ridenour WM, Callaway RM. The relative importance of allelopathy in interference: the effects of an invasive weed on a native bunchgrass. Oecologia. 2001;126(3):444–50. 28547460 10.1007/s004420000533

[44] He W, Feng Y, Ridenour W, Thelen G, Pollock J, Diaconu A, et al Novel weapons and invasion: biogeographic differences in the competitive effects of *Centaurea maculosa* and its root exudate (±)-catechin. Oecologia. 2009;159(4):803–15. 10.1007/s00442-008-1234-4 19219462

[45] Hahn MA, Buckley YM, Müller-Schärer H. Increased population growth rate in invasive polyploid *Centaurea stoebe* in a common garden. Ecology Letters. 2012. 10.1111/j.1461-0248.2012.01813.x 22727026

[46] Ricciardi A, Hoopes MF, Marchetti MP, Lockwood JL. Progress toward understanding the ecological impacts of nonnative species. Ecological Monographs. 2013;83(3):263–82.

[47] Callaway RM, Schaffner U, Thelen GC, Khamraev A, Juginisov T, Maron JL. Impact of *Acroptilon repens* on co-occurring native plants is greater in the invader’s non-native range. Biological Invasions. 2012;14(6):1143–55.

[48] Kaur R, Gonzales WL, Llambi LD, Soriano PJ, Callaway RM, Rout ME, et al Community impacts of *Prosopis juliflora* invasion: biogeographic and congeneric comparisons. PloS one. 2012;7(9):e44966 10.1371/journal.pone.0044966 22984595 PMC3440363

[49] Qin RM, Zheng YL, Valiente-Banuet A, Callaway RM, Barclay GF, Pereyra CS, et al The evolution of increased competitive ability, innate competitive advantages, and novel biochemical weapons act in concert for a tropical invader. New Phytologist. 2013;197(3):979–88. 10.1111/nph.12071 23252450

[50] Richardson DM, Ricciardi A. Misleading criticisms of invasion science: a field guide. Diversity and Distributions. 2013;19(12):1461–7.

